# Patterns of Work‐Related Musculoskeletal Disorders Among Dentists in Herat, Afghanistan: The Role of Ergonomics and Exercise

**DOI:** 10.1155/ijod/8602001

**Published:** 2026-09-14

**Authors:** Ozair Erfan, Mujibullah Jaheed, Gulbahar Take

**Affiliations:** ^1^ Department of Oral and Maxillofacial Surgery, Faculty of Stomatology, Herat University, Herat, Afghanistan

**Keywords:** Afghanistan, dentists, ergonomics, exercise, musculoskeletal disorders, occupational health

## Abstract

**Background:**

Work‐related musculoskeletal disorders (MSDs) are a major occupational health concern among dentists due to prolonged static postures and repetitive clinical tasks. Despite extensive global research, evidence from low‐resource settings such as Afghanistan remains scarce. This study aimed to determine the prevalence of MSDs and examine associated demographic, occupational, and ergonomic factors among dentists in Herat, Afghanistan.

**Methods:**

A cross‐sectional study was conducted among 223 dentists randomly selected from a sampling frame of 530 registered practitioners in Herat, Afghanistan. Data were collected between January and March 2026 using an adapted Dari version of the standardized nordic musculoskeletal questionnaire (NMQ) administered electronically. Descriptive statistics and bivariate analyses were performed, followed by multivariable binary logistic regression to evaluate selected factors associated with MSDs. Adjusted odds ratios (AORs) with 95% confidence intervals (CIs) were calculated. Statistical significance was set at *p* < 0.05.

**Results:**

The prevalence of MSDs was 50.7% during the preceding 7 days and 83.4% during the preceding 12 months. Overall, 84.3% of participants reported musculoskeletal symptoms during at least one of the two assessed periods, with the lower back and neck being the most commonly affected anatomical regions. In bivariate analyses, several demographic, occupational, and ergonomic variables showed statistically significant associations with specific MSDs outcomes, including physical activity, working position, adequacy of rest breaks, appropriate working distance, upper‐jaw working position, and selected gender‐related differences (*p* < 0.05). However, in the multivariable binary logistic regression model, none of the included occupational and ergonomic predictors remained statistically significant after adjustment. Therefore, the observed bivariate associations should be interpreted as exploratory rather than as evidence of independent predictors.

**Conclusion:**

MSDs were highly prevalent among dentists in Herat, indicating a substantial occupational health burden. Although several occupational and ergonomic factors were associated with MSDs in bivariate analyses, no independent predictors were identified after multivariable adjustment. These findings support the need for further prospective research and suggest that ergonomics education, appropriate working practices, workplace modifications, and regular physical activity should be further evaluated as potential strategies for reducing the burden of MSDs among dental professionals.

## 1. Introduction

Work‐related musculoskeletal disorders (MSDs) are among the most frequently reported occupational health problems affecting dental professionals worldwide. The nature of dental practice requires clinicians to maintain prolonged static and often awkward postures, perform repetitive hand and wrist movements, and sustain visual attention within a restricted working field. Over time, these occupational demands may impose cumulative biomechanical strain on the musculoskeletal system, particularly the neck, shoulders, back, and upper extremities, thereby contributing to the development of MSDs [1, 2].

The impact of MSDs extends beyond physical discomfort and may adversely affect dentists’ professional performance and overall well‐being. Persistent musculoskeletal symptoms may interfere with concentration, clinical performance, and the efficient delivery of complex dental procedures. In more severe cases, MSDs have been associated with absenteeism, increased healthcare utilization, and a reduced ability to continue clinical practice. These consequences highlight MSDs as an important occupational health concern within the dental profession [3, 4].

Physical activity has been investigated as a potentially modifiable factor associated with MSDs among dental professionals. Previous studies have reported that dentists who engage in regular physical activity, including stretching, strengthening, and aerobic exercise, may experience less musculoskeletal discomfort than those with lower levels of physical activity [2, 3]. Regular movement and physical conditioning may also help counteract some of the musculoskeletal strain associated with prolonged static and awkward working postures. Accordingly, physical activity may complement ergonomic measures in promoting musculoskeletal health among dental professionals [4, 5].

Both individual characteristics and occupational conditions have been associated with MSDs among dental professionals. Factors such as age, gender, and years of professional experience have been linked to musculoskeletal symptoms, while occupational exposures, including prolonged working hours, high clinical workload, and sustained static or awkward postures may also contribute to musculoskeletal strain [5]. Several occupational and ergonomic factors are potentially modifiable, particularly inadequate ergonomic awareness, inappropriate positioning of the dentist or patient, and limited use of ergonomic equipment, making them important targets for preventive strategies in dental practice [4–8].

Despite the growing global literature on MSDs among dental professionals, evidence from Afghanistan remains limited. Occupational conditions, access to ergonomic resources, dental training environments, and patterns of clinical practice may differ across countries, limiting the direct applicability of findings from other settings to Afghan dentists. Local evidence is therefore needed to describe the burden of MSDs and explore potentially associated occupational and ergonomic factors in this population. Such evidence may help inform context‐specific occupational health research and preventive strategies for dental professionals in Afghanistan.

Therefore, this study aimed to determine the prevalence of MSDs among dentists in Herat, Afghanistan, and to examine their associations with selected demographic, occupational, ergonomic, and physical activity‐related factors. By providing locally relevant evidence, the study may help inform context‐specific preventive and ergonomic strategies for dental professionals.

## 2. Materials and Methods

This cross‐sectional study was conducted among dentists practicing in Herat city, Afghanistan. According to the official registry of the Herat Directorate of Public Health, 530 dentists were registered at the time of the study. The required sample size was calculated using the Raosoft Sample Size Calculator (Raosoft Inc., Seattle, WA, USA), assuming a 95% confidence level, a 5% margin of error, and a 50% response distribution. Based on these parameters, the required sample size was 223 participants.

Participants were selected using simple random sampling through computer‐generated random selection from the official registry. A total of 223 selected dentists were invited to participate in the electronic survey. Data were collected over a 6‐week period, from January 23 to March 3, 2026. The questionnaire was administered electronically using Google Forms. Participants who did not initially complete the questionnaire were sent a reminder and were invited to complete it if they remained willing to participate. All questionnaire items applicable to each participant were set as required in Google Forms, preventing the submission of incomplete questionnaires. Conditional items, such as exercise frequency, were presented only to participants for whom they were applicable. All 223 invited dentists submitted complete questionnaires, resulting in a 100% response rate and no missing data for applicable items.

Eligibility criteria included completion of a recognized dental degree, official registration to practice dentistry, active dental practice in Herat city during the study period, and provision of informed consent. Dentists who had not completed a dental degree, were not officially registered to practice, were practicing outside Herat city, including surrounding districts or other provinces, or declined participation were excluded. No minimum duration of clinical experience was required, and no restriction was applied according to part‐time or full‐time practice status. The presence of systemic medical conditions was recorded but was not used as an exclusion criterion. Pregnancy status, recent traumatic injuries, and a history of MSDs predating dental practice were not specifically collected and therefore were not used as exclusion criteria.

The questionnaire comprised two sections. The first section collected demographic, health, occupational, and ergonomic information, including age, sex, marital status, height, weight, years of clinical practice, average daily clinical and administrative working hours, average number of patients treated per day, regular physical exercise, previous ergonomics education during undergraduate training, awareness of ergonomic principles, sitting posture during dental procedures, use of direct or indirect vision, and the operator’s working position during dental treatment. Body mass index (BMI) was calculated as weight in kilograms divided by height in meters squared (kg/m^2^). Participants were also asked whether they had any systemic medical condition and, if so, to specify the condition in an open‐ended item. All information was self‐reported.

The second section consisted of the standardized nordic musculoskeletal questionnaire (NMQ), which assessed musculoskeletal symptoms in nine anatomical body regions during the preceding 12 months and the preceding 7 days. The questionnaire also assessed symptom‐related consequences for each affected body region, including resting because of pain, reduced physical activity, temporary absence from work, and seeking medical consultation or treatment.

The NMQ was translated into Persian (Dari) and subsequently back‐translated into English with the assistance of an English‐language specialist. Before the main survey, the translated questionnaire was pilot‐tested among 15 dentists who were not included in the final study sample to assess its clarity, comprehensibility, and feasibility. Minor linguistic modifications were made based on participant feedback. The internal consistency of the adapted questionnaire was assessed using Cronbach’s alpha, which yielded a value of 0.84. To the best of our knowledge, no previously validated Afghan version of the NMQ has been published.

Ethical approval for this study was obtained from the Ethics Committee, Stomatology Faculty, Herat University, Herat, Afghanistan, on January 2, 2026 (Protocol No. 11). The study was conducted in accordance with the principles of the Declaration of Helsinki. Electronic informed consent was obtained from all participants before questionnaire completion. Participation was voluntary, and participant confidentiality and anonymity were maintained throughout the study.

Completed questionnaires were exported from Google Forms and analyzed using IBM SPSS Statistics version 26.0 (IBM Corp., Armonk, NY, USA). Descriptive statistics were used to summarize participant characteristics and study variables. Categorical variables were presented as frequencies and percentages, whereas continuous variables were summarized using medians and interquartile ranges (IQRs). Associations between categorical variables were assessed using the chi‐square test. A two‐sided *p*‐value  < 0.05 was considered statistically significant. No formal adjustment for multiple comparisons was applied; therefore, the bivariate analyses were considered exploratory, and individual *p*‐values were interpreted cautiously.

Multivariable binary logistic regression analysis was performed using the recoded total musculoskeletal pain variable as the dependent variable. The outcome was coded as 1 = presence of musculoskeletal pain and 0 = absence of musculoskeletal pain; therefore, the regression model estimated the odds of reporting musculoskeletal pain. The upper‐jaw working region, sitting position during dental treatment, and regular physical exercise were selected based on their associations in the bivariate analyses and their clinical relevance. All predictors were entered simultaneously using the Enter method. Adjusted odds ratios (AORs), 95% confidence intervals (CIs), and corresponding *p*‐values were reported.

Potential sources of bias were considered in the study design and interpretation. Because the data were collected using a self‐reported questionnaire, reporting and recall bias could not be excluded, particularly for symptoms recalled over the preceding 12 months. Simple random sampling from the official registry was used to reduce the potential for selection bias among registered dentists. However, because the sampling frame was restricted to officially registered dentists, unregistered or informally practicing dentists were not represented. The use of the standardized NMQ and pilot testing of the adapted questionnaire were intended to improve the consistency and comprehensibility of data collection.

## 3. Results

### 3.1. Participant Characteristics

**Figure 1:**
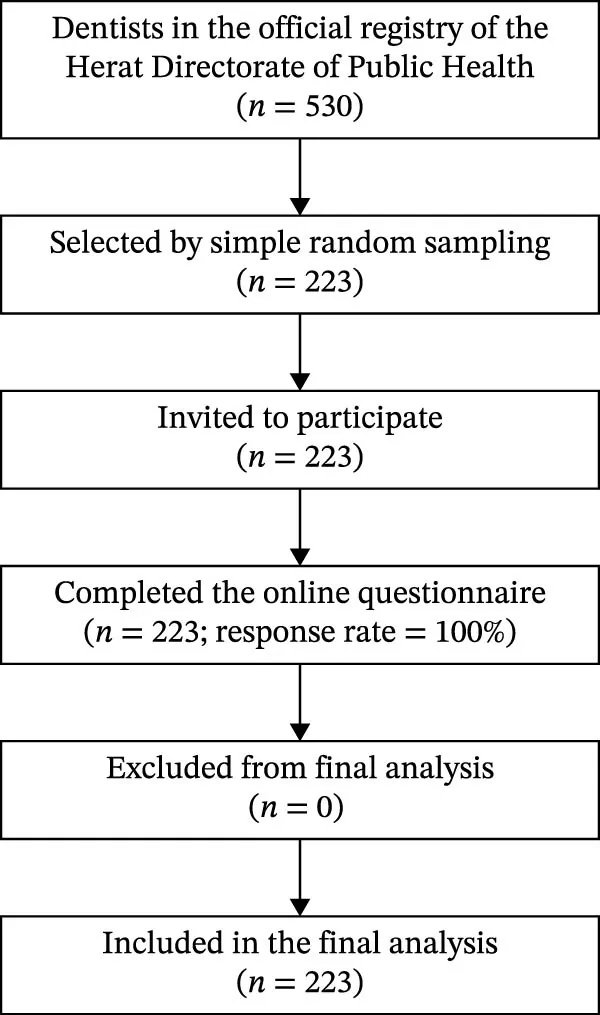
STROBE‐style flow diagram of participant selection, recruitment, and inclusion in the final analysis.

A total of 223 dentists were invited to participate in the study, and all completed the online questionnaire, resulting in a response rate of 100%. All submitted questionnaires were included in the final analysis. The participant selection and recruitment process is illustrated in Figure 1.

The study sample comprised 162 men (72.6%) and 61 women (27.4%). Regarding marital status, 173 participants (77.6%) were single and 50 (22.4%) were married. Most participants were right‐handed (90.1%), whereas 1.3% were left‐handed and 8.6% were ambidextrous. Systemic medical conditions were reported by 12 participants (5.4%), while 211 (94.6%) reported no systemic medical condition.

The median age of the participants was 29 years (IQR = 5 years). The median height was 172 cm (IQR = 11 cm), and the median weight was 73 kg (IQR = 18 kg). The median BMI was 24.90 kg/m^2^ (Q1–Q3: 22.49–27.68).

### 3.2. Prevalence of MSDs

**Figure 2:**
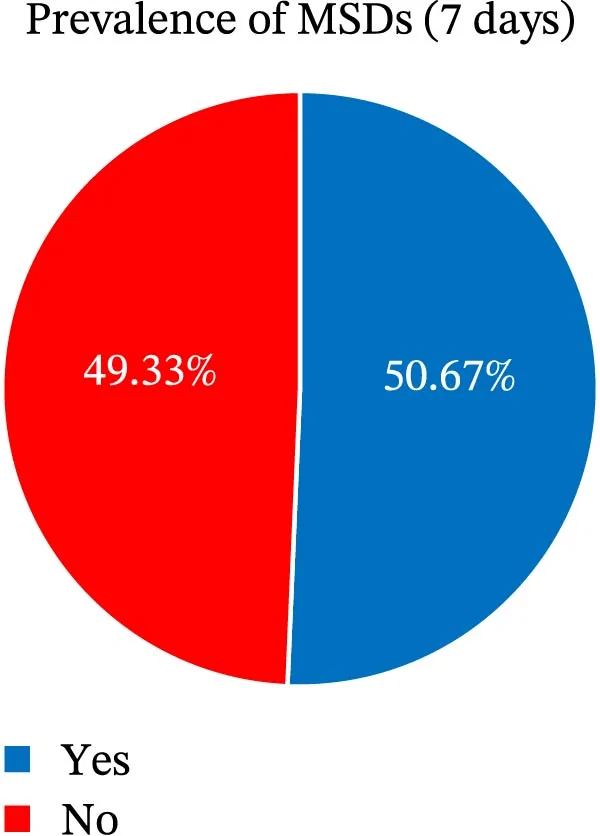
The prevalence of MSDs over the past week.

**Figure 3:**
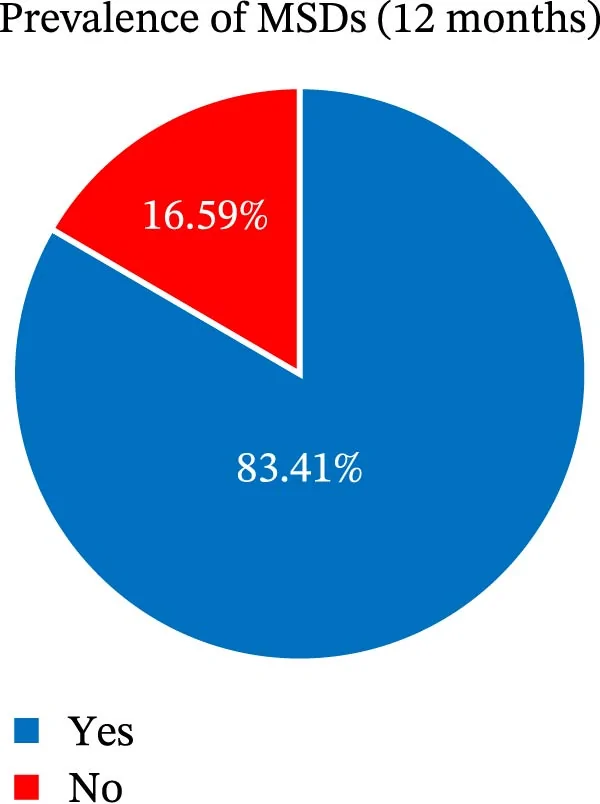
The prevalence of MSDs over the past year.

The prevalence of MSDs affecting at least one body region was 50.7% during the preceding 7 days (Figure 2) and 83.4% during the preceding 12 months (Figure 3). The 12‐month prevalence was higher than the 7‐day prevalence.

**Figure 4:**
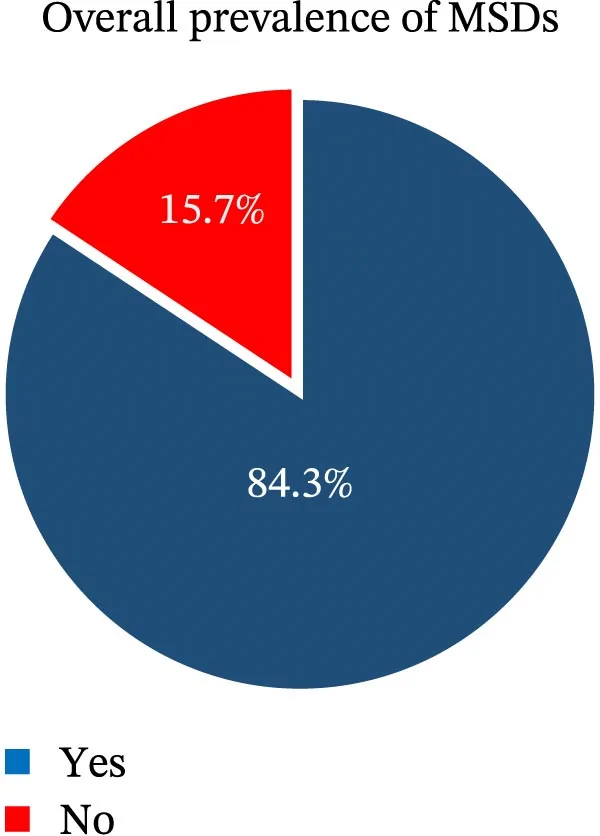
The overall prevalence of MSDs.

Overall, 188 of the 223 participants (84.3%) reported musculoskeletal symptoms during at least one of the assessed periods, whereas 35 participants (15.7%) reported no musculoskeletal symptoms during either period (Figure 4).

### 3.3. Physical Activity

Overall, 127 participants (57.0%) reported engaging in regular physical activity. In exploratory bivariate analysis, physical activity was significantly associated with overall MSD status (*p* = 0.043).

Exercise‐frequency data were applicable to 161 participants who reported engaging in physical activity. Among these participants, 27.3% reported exercising daily, 24.8% three times per week, 18.1% twice per week, 8.7% once per week, and 21.1% occasionally. The distribution of exercise frequency differed significantly by sex (*p* = 0.002). Among male participants with available exercise‐frequency data, the most commonly reported patterns were exercising three times per week (27.4%) and daily (25.8%), whereas female participants most commonly reported occasional activity (45.9%) and daily exercise (32.4%).

Participation in regular physical activity also differed significantly by sex. Regular physical activity was reported by 66.0% of male participants compared with 32.8% of female participants (*p*  < 0.001).

### 3.4. Distribution of MSDs by Body Region

**Figure 5:**
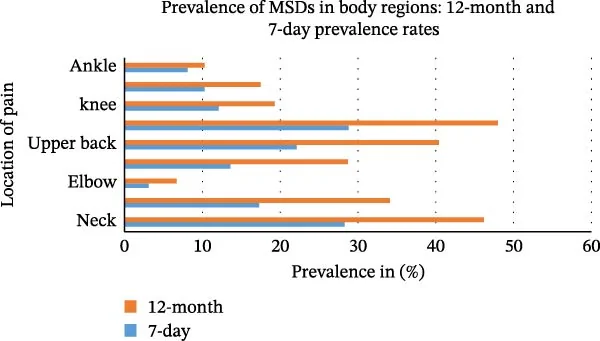
Distribution of MSDs in body regions; 12‐month and 7‐day prevalence rates.

The lower back was the most frequently affected anatomical region, with prevalence rates of 28.8% during the preceding 7 days and 48.0% during the preceding 12 months. The neck was the second most frequently affected region, with corresponding prevalence rates of 28.3% and 46.2%. The elbow was the least frequently affected region, with prevalence rates of 3.1% during the preceding 7 days and 6.7% during the preceding 12 months (Figure 5).

Significant sex‐related differences were observed for specific musculoskeletal outcomes. Male participants reported a significantly higher prevalence of knee pain during the preceding 7 days than female participants (*p* = 0.043). In contrast, female participants reported a significantly higher frequency of wrist‐related functional limitations, including the need to rest or reduce activity because of discomfort (*p* = 0.001).

### 3.5. Ergonomic Awareness and Occupational Characteristics

Most participants (78.0%) reported familiarity with ergonomic principles in clinical practice, and 60.5% reported receiving ergonomics training during their university education.

Regarding daily clinical working hours, 36.8% of participants worked fewer than 8 h, 30.9% worked 8 h, and 33.1% worked 10 h or more per day. Most participants worked exclusively in private practice (91.0%), while 8.5% worked in both private and public settings and 0.4% worked exclusively in the public sector. With respect to working posture, 69.1% reported working in both sitting and standing positions, 30.5% primarily worked while sitting, and 0.4% primarily worked while standing.

The duration of breaks between patients varied considerably. Irregular breaks were reported by 40.4% of participants, while 18.4% reported breaks of 6–10 min, 15.7% reported 5‐minute breaks, 8.5% reported breaks of 11–20 min, 3.1% reported breaks longer than 20 min, and 13.9% reported taking no breaks between patients. In exploratory bivariate analysis, break duration was associated with 7‐day MSD prevalence (*p* = 0.048).

Regarding working distance from the patient, 2.2% reported not maintaining an appropriate distance, 45.7% reported maintaining it to a limited extent, 27.8% to a considerable extent, and 17.5% completely, while 6.7% reported not paying attention to working distance. In exploratory bivariate analysis, working distance was associated with overall MSDs status (*p* = 0.036).

Detailed occupational and ergonomic characteristics, including daily patient load, work experience, ergonomics training, visual approach, and sitting posture during treatment, are presented in Table 1.

**Table 1:** Occupational and ergonomic characteristics of participating dentists (*N* = 223).

Variable	Category	*n* (%)
Working position	Sitting	68 (30.5)
	Standing	1 (0.4)
	Both sitting and standing	154 (69.1)
Place of employment	Public/official	1 (0.4)
	Private	203 (91.0)
	Both public and private	19 (8.5)
Daily clinical working hours	<8 h	82 (36.8)
	8 h	69 (30.9)
	10 h	40 (17.9)
	12 h	25 (11.2)
	>12 h	7 (3.1)
Daily administrative working hours	No administrative work	181 (81.2)
	<8 h	32 (14.3)
	8 h	7 (3.1)
	10 h	1 (0.4)
	12 h	2 (0.9)
Patients treated per day	<5	87 (39.0)
	5	52 (23.3)
	6–10	69 (30.9)
	11–20	11 (4.9)
	>20	4 (1.8)
Work experience in dentistry	1–5 years	119 (53.4)
	6–10 years	88 (39.5)
	11–20 years	12 (5.4)
	>20 years	4 (1.8)
Regular physical exercise	Yes	127 (57.0)
	No	96 (43.0)
Ergonomics taught during university	Yes	135 (60.5)
	No	88 (39.5)
Familiarity with ergonomic conditions in the clinic	Yes	174 (78.0)
	No	49 (22.0)
Sitting posture during treatment	Correct posture	121 (54.3)
	Incorrect posture	44 (19.7)
	Did not pay attention	58 (26.0)
Maintenance of appropriate distance from patient	Not maintained	5 (2.2)
	Maintained a little	102 (45.7)
	Maintained considerably	62 (27.8)
	Maintained completely	39 (17.5)
	Did not pay attention	15 (6.7)
Method of visualizing the target area	Direct vision	24 (10.8)
	Indirect vision	33 (14.8)
	Both	166 (74.4)
Level of ergonomics training at university	No training	37 (16.6)
	Little	97 (43.5)
	Moderate	64 (28.7)
	Extensive	16 (7.2)
	Complete	9 (4.0)
Perceived need for additional ergonomics training	Very little	34 (15.2)
	Little	19 (8.5)
	Moderate	25 (11.2)
	Much	18 (8.1)
	Very much	127 (57.0)

*Note*: Values are presented as frequency (percentage). All variables were self‐reported by participants.

### 3.6. Working Position Relative to the Patient

The dentist’s working position relative to the patient was significantly associated with MSDs prevalence during both assessed periods. Over the preceding 12 months, a significant association was observed between working position and MSDs prevalence when treating the first quadrant (*p* = 0.004) and the second quadrant (*p* = 0.013).

Similarly, during the preceding 7 days, working position was significantly associated with MSDs prevalence when treating the first quadrant (*p* = 0.022) and the second quadrant (*p* = 0.004).

### 3.7. Multivariable Logistic Regression Analysis

A multivariable binary logistic regression analysis was conducted to assess factors independently associated with musculoskeletal pain among the participating dentists. The variables included in the model were upper‐jaw working region, sitting position during dental treatment, and exercise status. All variables were entered simultaneously using the Enter method. The overall regression model was statistically significant (*p* = 0.049).

After adjustment for the other variables included in the model, dentists who mainly worked in the upper‐jaw region had higher adjusted odds of reporting musculoskeletal pain compared with the reference group (AOR = 2.01, 95% CI: 0.76–5.32). However, this association did not reach statistical significance (*p* = 0.158). Sitting position and exercise status also showed AORs greater than 1, indicating higher odds of reporting musculoskeletal pain; however, neither association was statistically significant after adjustment. Overall, none of the individual predictors included in the model reached statistical significance. The complete results of the multivariable analysis are presented in Table 2.

**Table 2:** Multivariable binary logistic regression analysis of factors associated with musculoskeletal pain among dentists.

Variable/comparison	Reference category	Adjusted OR (AOR)	95% CI	*p*‐Value
Mainly working in the upper‐jaw region	Lower‐jaw or other working region	2.01	0.76–5.32	0.158
Incorrect sitting position during dental treatment	Correct sitting position	2.20	0.77–6.31	0.137
No regular physical exercise	Regular physical exercise	1.89	0.88–4.04	0.103

*Note*: The dependent variable was the presence of musculoskeletal pain (1 = presence, 0 = absence). All predictors were entered simultaneously using the Enter method. An AOR greater than 1 indicates higher adjusted odds of reporting musculoskeletal pain compared with the stated reference category.

Abbreviations: AOR, adjusted odds ratio; CI, confidence interval.

## 4. Discussion

The present cross‐sectional study found a high prevalence of MSDs among dentists practicing in Herat, Afghanistan, with 84.3% of participants reporting symptoms during at least one of the two assessed periods. This prevalence is broadly comparable with the high prevalence reported among dental practitioners in Pakistan (87%) [8], India (92.4%) [9], and Germany (92%) [2]. Studies from Saudi Arabia have also reported substantial prevalence rates among practicing dentists and dental students [4, 7]. These comparisons should be interpreted cautiously because of differences in study populations, assessment instruments, definitions of MSDs, and recall periods. Nevertheless, broader international evidence confirms the substantial burden of MSDs in dentistry. A systematic review and meta‐analysis based on 89 prevalence estimates from 88 publications reported a pooled prevalence of 78.4% among dental healthcare providers [10], while another systematic review found that the annual prevalence of MSDs affecting at least one body region among dentists ranged from 68% to 100% [11]. A comparison of the prevalence of MSDs, commonly affected anatomical regions, and associated factors reported in the present study and selected previous studies is presented in Table 3.

**Table 3:** Comparison of musculoskeletal disorder prevalence, commonly affected regions, and associated factors across selected studies.

Study	Country and study population	Sample size	MSD prevalence	Most affected body regions	Ergonomic or associated factors/findings
Current study	Afghanistan (Herat); practicing dentists	223	7‐day: 50.7% 12‐month: 83.4% Overall: 84.3%	Lower back (48.0%), neck (46.2%), upper back	Working position relative to the patient; upper‐jaw working region; sitting position; exercise status; prolonged work hours and irregular rest breaks were commonly reported
AlSahiem et al. [7]	Saudi Arabia; dental students	462	12‐month: 87.0%	Neck (62%), lower back (56%), shoulders (46%)	Daily and prolonged clinical sessions, sitting position, clinic suitability for left‐handed operators, coping strategies, and physical activity
Al‐Huthaifi et al. [6]	Yemen; dental professionals and students	310	Overall: 72.6%	Neck (57.3%), lower back (48.9%), upper back (43.1%), shoulders (25.3%)	Low ergonomic knowledge, workload‐related pain, working conditions, patient volume, posture, and insufficient work breaks
Bracciale et al. [12]	Portugal and Italy; practicing dentists	341 Portugal: 170 Italy: 171	7‐day: 58.2%/56.7% 12‐month: 78.8%/81.9% (Portugal/Italy)	Neck (65.3%/61.4%), lumbar region (52.4%/50.9%), shoulders (49.4%/39.2%)	Longer practice time was a significant risk factor; workload, working position, breaks, and physical activity were also evaluated
Barsyte et al. [13]	Lithuania; dentists and oral hygienists	382	12‐month: 95.5%	Neck (65.7%), shoulders (59.7%), lower back (58.1%)	Physical inactivity and limited ergonomic knowledge were notable; weekly work hours and physical activity were assessed
Edrees et al. [14]	Germany; practicing dentists	576	Point prevalence: 59.7%	Multisite pain (719 pain sites among 344 dentists); neck pain was associated with higher chronification risk	Current pain—particularly leg and neck pain—was associated with a higher risk of pain chronification

Abbreviation: MSDs, musculoskeletal disorders.

The present study also revealed a marked difference between short‐term and long‐term MSDs prevalence, with 50.7% of participants reporting symptoms during the preceding 7 days and 83.4% during the preceding 12 months. A similar pattern was reported in India, where the corresponding 7‐day and 12‐month prevalence rates were 87.1% and 92.4%, respectively [9], and in Germany, where the corresponding prevalence rates were ~65.6% and 92.0% [2], respectively. The consistently higher 12‐month prevalence suggests that the longer recall period captures participants who experienced intermittent or previous musculoskeletal symptoms that were not necessarily present during the week immediately preceding the survey.

The lower back (48.0%) and neck (46.2%) were the most commonly affected anatomical regions in the present study. Similar patterns have been reported among dental professionals and students in Saudi Arabia, Yemen, India, Iran, Germany, and Pakistan, where the lower back, neck, shoulders, and upper back were frequently identified among the most affected regions [2, 4–9]. International systematic evidence also supports this pattern. One systematic review reported prevalence ranges of 29%–94.6% for the lower back, 25%–92.7% for the shoulders, and 26%–92% for the neck among dentists [11]. A global systematic review and meta‐analysis similarly identified the neck, back, lower back, shoulders, and upper back among the anatomical regions most frequently affected in dental healthcare providers [10]. These findings indicate that the cervical and lumbar regions are particularly susceptible to occupational musculoskeletal strain in dental practice.

The predominance of lower‐back and neck symptoms may be related to prolonged static or awkward postures, repetitive clinical movements, sustained flexion of the neck and trunk, and inappropriate positioning of the operator or patient during dental procedures [11, 15, 16]. These occupational conditions may increase mechanical loading of the cervical and lumbar regions and contribute to pain and functional limitations over time. However, because the present study had a cross‐sectional design, temporal or causal relationships between these occupational exposures and MSDs cannot be established.

Several occupational, ergonomic, and physical activity‐related variables were significantly associated with MSDs outcomes in the exploratory bivariate analyses. These included regular physical exercise, break duration, maintenance of an appropriate working distance, and the dentist’s working position relative to the patient. Comparable occupational and ergonomic factors have been evaluated among dental professionals in Pakistan, Yemen, Portugal, Italy, Lithuania, and Germany [6, 8, 12–14]. Previous systematic evidence has also identified awkward working postures, prolonged occupational exposure, high workload, and inadequate ergonomic practices as commonly reported factors associated with MSDs among dental professionals [11, 15]. Nevertheless, the bivariate findings of the present study should be interpreted cautiously because multiple statistical comparisons were performed without formal adjustment for multiplicity, potentially increasing the risk of Type I error. Furthermore, the observed associations did not remain statistically significant after multivariable adjustment.

An important finding of the present study was that 39.5% of participants had not received formal ergonomics education during their undergraduate dental training. This finding indicates a potential educational gap and suggests that ergonomics may not yet be sufficiently integrated into undergraduate dental education in this setting. A systematic review of ergonomic interventions reported that ergonomics education, magnification systems, dental‐chair modifications, and ergonomically designed instruments may improve working posture or musculoskeletal outcomes among dental professionals [17–20]. However, the available evidence remains limited, and additional high‐quality intervention studies with adequate follow‐up are required to determine the long‐term effectiveness of these measures. Ergonomics education should therefore include practical clinical application and periodic reinforcement rather than being limited to theoretical instruction.

More than 90% of participants in the present study were right‐handed, a distribution similar to that reported in previous studies involving dental professionals [2, 5, 21]. However, hand dominance was not identified as an independently associated factor in the present study. Accordingly, this finding should be regarded as a descriptive characteristic of the study population rather than evidence that right‐handedness itself contributes to the development of MSDs.

Although the overall multivariable binary logistic regression model was statistically significant, none of the individual occupational or ergonomic predictors reached statistical significance after adjustment. The AORs for mainly working in the upper‐jaw region, incorrect sitting position during dental treatment, and absence of regular physical exercise were greater than 1; however, all corresponding CIs included the null value. Therefore, the statistical significance of the overall model should not be interpreted as evidence that any individual predictor was independently associated with musculoskeletal pain. The high prevalence of musculoskeletal pain and the relatively small number of participants without pain may have reduced the precision of the adjusted estimates. Interrelationships among the included occupational and ergonomic variables may also have attenuated their individual effects. These findings should therefore be interpreted cautiously and should not be considered evidence of independent or causal relationships.

Overall, the findings demonstrate a substantial burden of MSDs among dentists practicing in Herat, particularly in the lower back and neck. Although several occupational, ergonomic, and physical activity‐related variables were associated with MSDs in the exploratory bivariate analyses, none remained statistically significant as an independent predictor after multivariable adjustment. The observed associations should therefore be regarded as hypothesis‐generating. Practical ergonomics education, appropriate positioning of the operator and patient, adequate rest breaks, improved workplace organization, and regular physical activity warrant further evaluation as potential preventive strategies in prospective and intervention studies.

## 5. Limitations

This study has several limitations that should be considered when interpreting the findings. First, the cross‐sectional design precludes establishing temporal or causal relationships between occupational and ergonomic factors and MSDs. Second, the data were collected using a self‐administered questionnaire and were therefore susceptible to recall and reporting biases, particularly for symptoms reported over the preceding 12 months. Third, although the adapted Dari version of the NMQ demonstrated acceptable internal consistency and was pilot‐tested before data collection, it has not been formally validated in the Afghan population.

Although participants were selected using simple random sampling, the sampling frame was limited to dentists officially registered with the Herat Directorate of Public Health. Consequently, unregistered or informally practicing dentists may not have been represented. Furthermore, the study was conducted only among dentists practicing in Herat city, which may limit the generalizability of the findings to dentists working in other regions of Afghanistan or in different occupational settings.

The high prevalence of musculoskeletal pain resulted in a relatively small number of participants without pain, which may have reduced the precision and statistical power of the multivariable analysis to identify independent associations. Pregnancy status, recent traumatic injuries, and MSDs that existed before entering dental practice were not specifically assessed and therefore could not be evaluated as potential confounding factors. Finally, multiple statistical comparisons were conducted without formal adjustment for multiplicity, potentially increasing the risk of Type I error. Accordingly, associations with *p*‐values close to the conventional significance threshold should be interpreted cautiously and confirmed in future longitudinal studies with adequate statistical power.

## 6. Conclusion

This study demonstrated a high prevalence of MSDs among dentists practicing in Herat, Afghanistan, with the lower back and neck being the most commonly affected anatomical regions. Although several occupational, ergonomic, and physical activity‐related factors were associated with MSDs in the exploratory bivariate analyses, none remained statistically significant after multivariable adjustment. These findings indicate a considerable occupational health burden but do not establish independent or causal relationships between the examined factors and MSDs.

Despite the relatively high level of reported ergonomic awareness, MSDs remained common, suggesting that awareness alone may not be sufficient to ensure effective ergonomic practice or prevention. Practical ergonomics training, appropriate operator and patient positioning, adequate rest breaks, improved workplace and equipment design, and supportive work organization should be further evaluated in prospective and intervention studies as potential strategies for reducing the burden of work‐related MSDs among dental professionals.

## Author Contributions

**Ozair Erfan:** conceptualization, methodology, investigation, data curation, formal analysis, supervision, project administration. **Mujibullah Jaheed:** investigation, validation, writing – review and editing. **Gulbahar Take:** writing – original draft, writing – review and editing.

## Funding

This study did not receive any specific funding.

## Disclosure

All authors have read and approved the final version of the manuscript. Ozair Erfan, the corresponding author, had full access to all of the data in this study and takes complete responsibility for the integrity of the data and the accuracy of the data analysis.

## Conflicts of Interest

The authors declare no conflicts of interest.

## Data Availability

The authors confirm that the data supporting the findings of this study are available within the article. Additional data are available from the corresponding author upon reasonable request.
